# The Mediating Role of Organisational Identification between Psychological Contract and Work Results: An Individual Level Investigation

**DOI:** 10.3390/ijerph19095404

**Published:** 2022-04-28

**Authors:** Anna Rogozińska-Pawełczyk, Katarzyna Gadomska-Lila

**Affiliations:** 1 Department of Labour and Social Policy, Faculty of Economics and Sociology, University of Lodz, 90-136 Lodz, Poland; 2 Institute of Management, University of Szczecin, 70-453 Szczecin, Poland

**Keywords:** psychological contract, relational psychological contract, transactional psychological contract, organisational identification, work results, HR professionals

## Abstract

The aim of this article is to identify the relationship between the fulfilment of relational and transactional psychological contracts and work results, taking into account the mediation effect expressed in organisational identification. The empirical research was conducted on a group of 402 HR professionals responsible for designing and implementing HR practices in one of the leading companies of the Polish energy sector. Hypotheses were tested using the partial least squares structural equation modelling technique (PLS-SEM). Based on our research, we found that the implementation of both relational and transactional psychological contracts positively influenced the results achieved by HR professionals, both directly and indirectly, through the mediating role of organisational identification. The results indicate that the relationship between the psychological contract and work results is stronger when mediated by organisational identification. It plays an important role, especially in relation to the transactional contract. The collected results lead to the conclusion that organisations, wishing to increase the level of work results achieved by HR professionals, should as much as possible fulfil the expectations of employees and meet the commitments made to them within the framework of the established psychological contract. The study makes an important contribution to the understanding of the “priority” importance of organisational identification in enhancing the efforts of HR professionals to deliver work results that benefit both employees and the organisation.

## 1. Introduction

In recent years, the labour market has been changing as a result of a number of economic, technological and social developments, such as increased global competition, the advancement of technology or increased flexibility of labour relations. They are reflected at the organisational level, influencing the employment and working conditions of employees [1,2,3]. Consequently, employees expect the organisation to meet a wide variety of obligations within the framework of a formal and an informal contract [4]. The psychological contract plays a very important role. This is because it influences the attitudes and behaviour of employees, and also the performance of the organisation [5]. It refers to various aspects of employee relations in an organisation [6] and the consequences of accepting and meeting mutual obligations to achieve the organisation’s goals [7]. It can help employers understand and predict employee behaviour, encourage employees to become more committed and contribute more to achieving the company’s goals [8]. Therefore, many researchers have studied the concept of psychological contract [9,10,11]. However, there is little research on other variables as mediators in the study of psychological contract. For example, there is little research on the relationship between psychological contract and work results considering organisational identification as a mediating variable. Meanwhile, organisational identification appears to be an important consequence of fulfilling, or violating, psychological contracts. It is therefore important to ask whether and how organisational identification mediates the relationship between psychological contract and work results, especially if we consider the two types of psychological contracts—relational and transactional. Based on the assumptions of social exchange theory [12,13], widely used in HRM [14] and organisational behaviour research, we tried to explain the nature of these relationships. We analysed them in relation to HR professionals responsible for managing human resources in organisations. The HR industry is developing intensively every year, and its representatives perform important functions in the structures of modern organisations. HR professionals are the link between management and employees, on the one hand helping managers at various levels to achieve the objectives for which employees are responsible, but also helping employees to develop specific competencies and attitudes and behaviours by designing and implementing HR practices. Their role seems to be particularly important, as it comprises the responsibility for the selection of methods, tools and practices of human resources management, which allow to shape the attitudes and behaviours of employees [15,16]. The changes taking place in the environment of contemporary organisations, as well as in the organisations themselves, make it necessary for HR professionals to learn how to respond faster to these changes, how to support their business entity even more effectively, and how to set an example to other employees with their attitudes and behaviours. Given this important role of HR professionals for organisations, the issue of studying the attitudes and behaviours of this group of employees is of particular importance. Meanwhile, there is a clear gap in empirical research on the mediating role of organisational identification between the psychological contract (especially in the distinction between transactional and relational) and the work results of HR professionals. The implementation of this type of research will therefore reveal the mechanism of the emergence of employee behaviour resulting from the fulfilment of the concluded psychological contracts, and thus contribute to filling the identified research gaps.

## 2. Theoretical Framework and Hypothesis Development

### 2.1. Psychological Contract

Going beyond the framework of the formal employment contract, while taking into account some of the subjective and normative elements involved in managing people [17], the psychological contract is a key determinant of employee attitudes and behaviours [18]. The subjective nature of psychological contracts is related to individual beliefs that arise in a specific social context and are shaped by the employee’s interactions with the employer [19,20]. Definitions of psychological contract most often emphasise expectations [21,22], which are of a promised and reciprocal nature [19], or obligations in the form of contracts, promises or other types of social, moral or legal requirements that compel one to follow or avoid a certain course of action [23]. In the authors’ understanding, a contract is mainly about expectations, the fulfilment or non-fulfilment of which gives rise to various consequences—it translates into different attitudes and behaviours of employees and directs the organisation’s activities. According to Wellin [24], the psychological contract has a positive impact on employees and employers. It enables a better understanding and prediction of people’s behaviour in the organisation, influences the increase of employees’ motivation level, as well as the intensification of activities that constitute the realisation of the organisation’s strategic goals. The terms of a psychological contract are not written down, defined, negotiated or discussed, but can be reformulated by the context, which implicitly or explicitly conveys a future commitment or intention [25]. The most common division in the literature is between relational and transactional contracts [2,18,26,27], although some authors, including Rousseau [28], enrich this division with transitional and balanced contracts. The dominant division, however, is between relational contracts, representing socio-emotional goods, and transactional contracts, representing the material interests of workers.

A transactional contract is purely economic, monetary and material in nature [29,30]; hence, the precise definition of inputs and benefits is important here. The duration of this type of contract is relatively short and limited, breaking is easy, the expected outcomes are precisely set and the responsibility for achieving them is clearly defined [31,32]. This means that transactional psychological contract-oriented employees view their organisation primarily as a source of income and a workplace [29]. Their contribution is limited due to low levels of attachment to the organisation. Consequently, employees with a transactional psychological contract are more likely to leave their jobs because they tend to treat their current position as a starting point for their future career [18]. Under this type of contract, the commitment on the part of the organisation is to create conditions conducive to individual professional development, which consequently promotes the employee’s career potential in the labour market.

The relational contract is based mainly on the exchange of employee loyalty to the organisation and commitment to its interests in return for job security and the opportunity to pursue a career within the organisation. This type of contract assumes employment in a long-term, indefinite perspective, cooperation based on mutual trust and loyalty, a relatively loose relationship between work performance and remuneration, a community of norms and values observed, both by employees and superiors, and group responsibility for achieved results [31,32,33]. The career creation of employees in a relational psychological contract rests primarily with the organisation and involves the creation of opportunities for vertical and horizontal career advancement, the development of a clear employee promotion plan and the development of full-time and long-term employment [34].

Activities currently undertaken in organisations, such as restructuring, downsizing or outsourcing, in response to international competition or technological progress, make it increasingly difficult for organisations to fulfil psychological contracts [35,36]. Building on the assumptions of social exchange theory [37], it is therefore worth attempting to explain the consequences that may arise in response to a breach of a psychological contract. This is currently the dominant paradigm in explaining the nature of organisational relationships [12,13]. Social exchange theory focuses on relationships understood as an exchange of resources between two parties. If one party provides a benefit, the other party feels obliged to do the same. Thus, based on these assumptions, it is reasonable to assume that fulfilling a psychological contract will positively affect the employer–employee relationship because it instils in employees a sense of obligation to care about the organisation and help achieve its goals [38]. On the other hand, when organisations fail to deliver on their promises, it is to be expected that employees will reduce their contribution to the organisation and adopt negative attitudes towards it [35]. These effects are particularly reflected in the level of organisational identification [26].

### 2.2. The Mediating Role of Organisational Identification

Organisational identification is the extent to which employees define themselves in terms of what they believe the organisation represents. It involves a perception of ‘oneness’ with or belonging to the organisation [39]. Organisational identification can also be seen as the process by which the goals of the organisation and the goals of the individual become increasingly integrated or compatible [25]. When employees strongly identify with their organisation, they satisfy their needs for belonging and affiliation [40,41]. Indeed, organisational identification is a kind of glue that sustains the relationship between employee and employer [42]. Research [39,43] has shown that employees with a strong sense of organisational identification are more likely to exhibit positive behaviours desired by the organisation. The more employees identify with their organisation, the more they will be willing to devote their efforts to it and engage with it [44,45], and less likely to express their intention to leave their job [46]. Therefore, employees who identify with the organisation are more likely to achieve their goals, collaborate and generally bring positive performance behaviours to the organisation, including beyond what is required (i.e., citizenship behaviours).

Thus, fulfilling the requirements of the psychological contract appears to be an important factor in employees’ organisational identification [47]. On the other hand, breach of contract reduces organisational identification because it involves employees’ perceptions that their needs are not being met. Numerous studies, including Epitropaki [42], Gibney, Few, and Scott [35] have confirmed the negative relationship between contract breach and organisational identification. When employees begin to perceive that employers are not living up to their commitments, this results in low levels of: productivity [48], trust [49], commitment [50] and organisational identification [42], and causes high levels of intention to leave the company [51]. It is therefore reflected in broadly defined work results. Hence, exploring what the relationship between psychological contract, especially its different types, and work outcomes is, and identifying the role of organisational identification, may lead to new theoretical explanations of these relationships.

### 2.3. Conceptual Model

The psychological contract is an important factor contributing to employee attitudes and behaviour, in relation to job satisfaction, organisational commitment [16], organisational trust, absenteeism, turnover intention [52,53,54], as well as to employees’ organisational identification and employee performance [55,56,57]. The analysed research model takes into account the construct of psychological contract fulfilment existing when the employer fulfils promises (transactional or relational type promise), which conditions the fulfilment of promises declared by the employee (transactional or relational type promise). The theoretical framework for specifying the scope of psychological contract fulfilment is the social exchange occurring between employee and employer and the accompanying norm of reciprocity [28]. The concept of the psychological contract introduces two distinctive types of psychological contracts in employment relationships, specifically (discussed in Section 2.1) the relational psychological contract and the transactional psychological contract [58]. Key differences between the two types of psychological contracts include the duration of the employment contract (short-term vs. long-term), the degree of specificity (highly specific vs. flexible), the exchange of resources (material vs. non-material), and the conditioning on performance and rewards (highly specific vs. low specific) [17,58,59]. Although other researchers have distinguished more than two types of psychological contracts, the most accepted approach to understanding types of psychological contracts is the two-dimensional approach, namely, transactional and relational psychological contracts [18,26,58].

The second construct examined in the hypothetical model is work results, which refer to an employee’s effectiveness in fulfilling his or her basic job duties or job role [5,55,60], and is thus defined as self-perception of performance. Thus, work results reflect how an employee performs within the demands of organisations. Social exchange theory, which emphasises reciprocity, also provides a theoretical basis for understanding how HR professionals, through the fulfilment of psychological contracts, initiate specific attitudes and organisational behaviours, thereby influencing the level of organisational performance and self-perception of performance, understood as work results [61,62]. This is because employees respond to their perceptions of whether there is a discrepancy between how they are actually treated by organisations and what they are promised in exchange for fulfilling their job obligations [55]. Employees will not adequately discharge their obligations to the organisation if they feel the organisation is not fully meeting its obligations [11]. In turn, when employees perceive that their organisation provides them with all the obligations it has promised them, they will begin to strengthen the psychological contract and increase their contributions to the organisation [14].

Organisational identification is the third construct that structures the hypothesised research model, determining the extent to which employees identify with their perceived representation of the organisation or simply their perceived unity with or membership of the organisation [63]. When employees strongly identify with their organisation, their needs for loyalty and identification with the organisation are met [17]. Employees integrate employer identity into their own social identity when they identify with the organisation [35].

Organisational identification also becomes a crucial element of the relationship between employees and the organisation, and thus linked to social exchange theory, by determining the level of perceived membership in the organisation [42]. Research has revealed significant effects of psychological contract breach and implementation on organisational identification [64]. The conceptual framework of organisational identification indicates that psychological contract breach weakens employees’ perceptions of organisational identification because it is directly related to employees’ individual perceptions of unfulfilled needs [55]. Breach of psychological contract discourages employees from investing their effort in the organisation and their sense of organisational identification is significantly reduced [65]. In turn, fulfilling the psychological contract is an important factor in increasing employees’ perceived organisational identification [66]. This is because fulfilling the psychological contract reduces employees’ uncertainty, thus motivating them to identify themselves organisationally [47].

**Figure 1:**
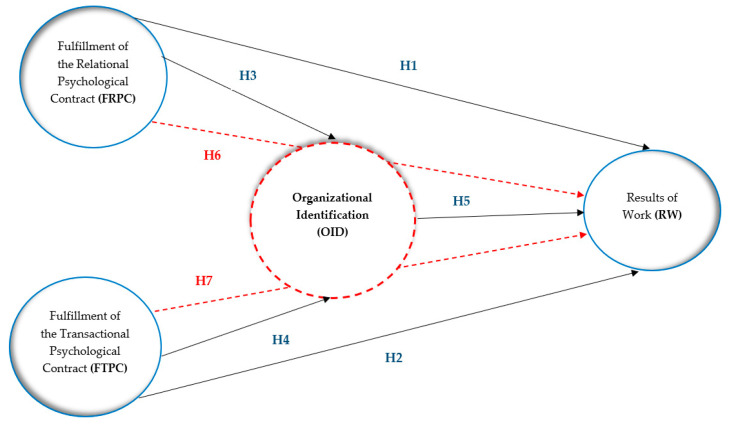
Research model of relationships between the fulfilment of the relational and transactional psychological contract and HR professionals’ work results and the mediating effect of organisational identification.

The increased pace of economic change will not only change the demand for labour, but also change the nature of work and human capital, including HR professionals, due to underlying trends in technology and automation of work [67,68]. HR professionals are a unique group whose role is to shape the required attitudes and behaviours of employees by designing and implementing appropriate HR management tools. This professional group is in a way a confirmation of the effectiveness of the HR tools applied, and at the same time a reference point for modelling the behaviour of other employees. In this unique context, whether and how fulfilling the psychological contract motivates HR professionals to perform their job duties and professional role to the highest possible standard, thereby achieving the best possible work results, has not yet been investigated. Furthermore, there is no clear evidence on whether HR professionals perform their job duties. Do they perform them only to achieve measurable economic outcomes or more to sustain long-term relationships? It therefore becomes crucial to answer the question of whether the fulfilment of the transactional psychological contract plays as important a role as the fulfilment of the relational psychological contract in the achievement of work results by HR professionals. A research hypothetical model was created to show the relationship between the tested variables is illustrated in Figure 1.

To provide an answer to the research question, we decided to use a set of seven hypotheses. Thus, we propose the following hypotheses:

**Hypothesis** **1** **(H1).** *The fulfilment of a relational psychological contract is positively related to the achieved work results of HR professionals in the surveyed organisation.*

**Hypothesis** **2** **(H2).** *The fulfilment of a transactional psychological contract is positively correlated with the achieved work outcomes of HR professionals in the surveyed organisation.*

**Hypothesis** **3** **(H3).** *The fulfilment of relational psychological contract is positively related to organisational identification.*

**Hypothesis** **4** **(H4).** *The fulfilment of the transactional psychological contract is positively associated with organisational identification.*

**Hypothesis** **5** **(H5).** *Organisational identification is positively linked to work results achieved by HR professionals in the surveyed organisation.*

**Hypothesis** **6** **(H6).** *Organisational identification mediates the relationship between the fulfilment of the relational psychological contract and the work results achieved by HR professionals in the surveyed organisation.*

**Hypothesis** **7** **(H7).** *Organisational identification is a mediator in the relationship between the fulfilment of the transactional psychological contract and the work results achieved by HR professionals in the surveyed organisation.*

## 3. Methods

### 3.1. Procedure

The main objective of the quantitative study was to explore the mechanism driving HR professionals’ achievement of work results (RW) from the perspective of the implementation of relational and transactional psychological contract (TPCF/ RPCF), in which organisational identification (OID) is considered as an important predictor of the overall process. The implementation of the research process consisted of the following stages:

The first stage of the research process was to develop an analysis of national and international literature on the concept of psychological contract and the way in which the fulfilment of transactional and relational psychological contract influences the work results achieved by HR professionals, the mediating role of organisational identification, and the relationship between the fulfilment of both types of contract and work results. The analysis the literature on the subject (desk research, web research) constituted the substantive basis for the implementation of the primary research, providing analytical framework information, enabling the correct formulation of the research problems undertaken within the framework of the own research.

The second stage of the research process consisted in developing research hypotheses from the conducted literature analysis and constructing a hypothetical research model. This stage allowed the identification of variables relevant to the process under study, deepening the understanding of these variables and determining the postulated relationships between them.

The third step in the research process was to design a measurement instrument to collect data on the main constructs within the proposed hypotheses. The measurements for each construct were developed based on the literature review and the specific questions within the survey questionnaire were adapted from validated instruments used in other studies and modified to fit the proposed research context.

The next step was to identify potential respondents and choose the method of data collection. The research was carried out using the computer-assisted telephone interview technique (CATI), ensuring anonymity in the procedure.

The fifth step of the quantitative component was the analysis of the collected statistical data obtained from the quantitative survey using a number of statistical analysis methods, including exploratory factor analysis EFA, confirmatory factor analysis CFA and structural equation modelling SEM.

In step six, the results obtained from the quantitative CATI survey were analysed in detail. For this purpose, the obtained research material was synthesised and the research hypotheses were verified. Next, the results of the survey at the employee and organisational level are discussed, while presenting the research limitations.

### 3.2. Measures

Three constructs were used to represent the factors highlighted in the hypothetical model: Fulfilment of the relational and transactional psychological contract, Organisational identification and Work results. To measure each construct, questions were adapted from validated instruments used in previous studies [19,55,69,70,71,72,73,74] and modified to fit to the research context. The survey questionnaire was designed to ensure that all participants understood the questions and anonymity was ensured. A battery of tests aggregated to a single questionnaire was used to collect empirical material. The variation of some problem areas included in the statements and questions was due to substantive and methodological reasons. In terms of content, the aim was to capture the relationship between the fulfilment of two types of psychological contract and work results with the mediating role of organisational identification. Methodologically, the collection of knowledge regarding the enhancement of work results as a measure of employee self-perceptions of job performance allowed for the verification of the consistency of the data obtained and the fulfilment of the condition of collecting data from multiple sources (multisource of data), which is recommended by researchers as an important condition for the accuracy of the study [75]. This becomes important when respondents describe their own behaviours and attitudes. Prior to the study, some tools required cultural adaptation. For this purpose, one of the procedures of tool adaptation [76] was used: translation and post-translation from the Polish version of the text to the original (English) version, in order to faithfully translate the methods used. The translated questionnaires were evaluated by competent judges, who were three independent experts in the field of: HRM, work psychology and statistical methods. The equivalence criteria of the questionnaires were also taken care of in the form of: facial equivalence, psychometric equivalence, functional equivalence and reconstruction fidelity [76].

The following set of diagnostic tools was used to measure the constructs:

*Fulfilment of the relational and transactional psychological contract* was assessed by adapting 20 items diagnosing two subscales: transactional and relational contract fulfilment [19,69]. Classification was based on the categorisation system used by Thompson and Hart [70]. The variables assigned to the relational psychological contract included social elements such as a good atmosphere in the workplace, values related to the social responsibility of the organisation and the relationship between the employee and the employer (e.g., job security and safety or honesty of the employer).The variables classified under the transactional psychological contract category included economic elements i.e., salary, its level and composition and the distribution of profit due to the employee’s contribution and elements that may be a source of future income e.g., opportunities for personal development or promotion.

The two subscales consisted of 10 items of transactional and relational promises respectively. An example item on transactional fulfilment of a psychological contract is: “*Training me only for my current job*” or “*It can terminate my employment any time*”. Similarly, relational fulfilment of the psychological contract was measured by 10 items such as: “ *I can make decisions with my interests in mind” or “The company is concerned about my personal welfare*”. The response system was based on a 5-point Likert scale, where 1 is “*not fulfil at all*” and 5 is “*fulfil completely*”.

*Organisational identification* was measured using six items created based on the description of the concept of organisational identification defined as related to the sense of belonging and the connection an employee has with the organisation [66] and the findings of a study by Ashforth and colleagues [72]. The questions in the survey questionnaire regarding organisational identification refer to when an employee fully or partially identifies him/herself by incorporating the organisation’s identity into their own [73].

An example question used in the survey questionnaire is: “*I am proud to be an employee of my organization*”. When assessing organisational identification, respondents were asked to indicate the extent to which they agreed or disagreed with the statements on a five-point Likert scale from 1—“*strongly disagree*” to 5—“*strongly agree*”.

*Results of work*—The last section of the survey questionnaire was devoted to exploring HR professionals’ personal experiences of achieving work results. The literature emphasises that an increase in organisational performance levels is made possible by individual employee performance and the results of work achieved by employees. The concept of performance refers to the extent to which an employee achieves the goals set by the organisation. Employee results of work, on the other hand, refers to the level of contribution or productivity of an employee that plays a significant role in enhancing organisational success [74]. Thus, when defining job performance, attention is paid more to the subjective aspects and approaches referring to the undertaking of certain attitudes, behaviours and work-related roles.

Achieved work results as a theoretical construct have not been directly observed so far and therefore constituted lateral variables in the study. The subject of measurement in this study will therefore be empirical indicators in the form of work results, i.e., effectiveness, efficiency, development, innovativeness and quality of work, which are an integral effect of fulfilling the psychological contract. For the purposes of this methodological procedure, five items were adopted with reference to the conceptualisation of employee performance measurement (in the sense of work results) by Drucker and Turnley with colleagues [55,77]. In assessing the items in this part of the questionnaire, the respondent was asked to follow the instructions given: “*Please circle the answers that correspond to your experiences at work*”. Sample items were: “*I perform my job duties carefully, professionally and efficiently*” or “*I continuously develop my competences necessary to meet future opportunities and challenges*”. A 5-point Likert scale was suggested as a way of answering, where 1 means “*strongly disagree*” and 5 is “*strongly agree*”.

The questionnaire used in the quantitative study, which includes all of the constructs described above, along with their assigned items, is included in Appendix A.

### 3.3. Participants

The study focused on HR professionals as it related to the achievement of work results, which depend on the perception of fulfilling the assumptions of the psychological contract related to the performance of HR tasks. This is because the concept of the research is based on the analysis of the mechanism that drives the achievement of work results by HR professionals who, through the realisation of positive relationships with their superiors, are the providers of expected HR actions in companies.

The arguments cited above determine the way in which the population of the study is defined, taking into account all employed HR professionals in the organisation under study. This study covered the individual employee level. This is related to the adopted definition of the psychological contract, which refers to the two parties to the contract involving the psychological contract: the employee and the employer. The employee, as one party to the contract, is relatively easy to identify because the psychological contract is perceived and maintained at the individual level. Employees, on the other hand, perceive the employer as the other party to the contract, through the prism of the organisation, defining ‘employer’ as the overall picture of the actions of supervisors and managers and the signals coming from the organisation in the form of HR practices and applicable company documentation. In this view, employees attribute characteristics to the employer that indicate an anthropomorphisation of the organisation [78].

The quantitative study was the main stage of the procedure carried out in order to empirically verify the hypothetical model. The survey covered 402 respondents, who constituted 100% of the employees in the HR department of the company under study. It used the technique of computer-assisted telephone interviewing (CATI). The research was preceded by a pilot study, which included the verification of the research tool. The final research tools were also verified by conducting a factor analysis and calculating reliability parameters. The data collected from each interview were analysed for the questionnaire path, which was guided by a script, as well as for the consistency of the tool. A self-constructed survey questionnaire and purposive sampling [79,80] were used.

Table 1 shows the demographics of the study participants. Overall, 64.9% of the participants were female and only 35.1% were male. This ratio indicates that the HR department in the energy industry, as in other industries [54], is dominated by women.

Half of the total group of respondents were under 39 years old (50.0%) and almost three quarters of the surveyed group had a master’s degree (71.4%). In terms of length of service with their current company, 35.8% of respondents said up to 10 years, while 61.5% of respondents surveyed had 10 years or more of total work experience. The distribution of positions in the professional hierarchy has the standard appearance of an inverted triangle. The majority of survey participants are employed as HR assistants and senior assistants (74.8%) and a minority as managers (20.0%) and executives (5.2%).

### 3.4. Data Analysis Methods

Structural equation modelling was used to analyse the collected data using the PLS method in WarpPLS 7.0 [81,82]. This is a confirmatory statistical analysis that aims to maximise the explained variance of the dependent variables by the predictors. Structural equation analysis, using the Partial Least Squares (PLS) method, was conducted in two steps. In the first step, latent variables were created [83] with the help of confirmatory factor analysis performed on observable variables. The formatted variables were used in a multivariate path analysis leading to a structural model. During structural equation modelling with the PLS method, multivariate regression analyses are performed in an iterative flow-a path structural model of the modelled relationships between variables is calculated. The path structural model returns information about the significance, sign and strength of predictor relationships with dependent variables.

The most important criterion for evaluating a path model is the generalised predictive power of the dependent variables. In the analysis, the PLS algorithm was applied using the information that the measurement model of the variables is a reflective model. The reflective measurement model was calculated based on the assumption that the latent variable affects the variability of observable variables that are correlated with each other and measured with their natural measurement error [83]. Standard errors and statistical significance were estimated through the Stable3 method proposed by a software developer [84]. It ensures that errors are computed in the course of exponential smoothing rather than bootstrapping (repeatedly drawing observations from a sample with returning them) [85]. The analysis predicted linear relationships between variables.

The tested model included measurement of all analysed variables. Diagnostic statistics of the measurement model and the structural model showed a very good fit of the data to the measurement model (external) SRMR = 0.09, SMAR = 0.08. Moreover, analysis of the overall predictive power of the structural model (internal) showed that it had a strong predictive power GoF = 0.51 [86]. The analysis also showed that the variables in the structural model were not strongly collinear with each other AVIF = 1.94. It was also observed that there was no total collinearity between the study variables AFVIF = 2.35. The model quality assessment statistics are presented in Table 2.

## 4. Results

In order to analyse the results of the measurement model in detail, a confirmatory factor analysis matrix of the variables conceptualised in the factor model was calculated. The analysis showed that all test items of the questionnaire were strongly and significantly associated with their factors.

Composite reliability (CR) coefficients were calculated to check the reliability of the measurements. All factor loadings in the measurement model exceeded 0.7 and factor analysis showed that all measurements had a high level of measurement accuracy CR/α > 0.75 [87,88]. The values of the extracted average variance (AVE) were higher than 0.50. Moreover, all square roots of the AVE are also higher than the interconstruct correlations. Thus, we confirmed the convergent validity of the scales [89,90]. Descriptive statistics were also calculated for the study variables and correlation analysis was performed, which in each case proved to be positively correlated and statistically significant. The results are presented in Table 3. The values obtained and the directions of the relationships can be considered as consistent with the predictions. However, in order to analyse the direct and indirect relationship between the variables, it was necessary to carry out analyses using structural equation modelling.

In addition to hypotheses H1–H5 which assume a direct and positive effect of the studied variables, hypotheses H6–H7 indicating an indirect positive effect, were additionally presented. It was assumed that the mediating effect between the fulfilment of the (relational and transactional) psychological contract and the work results achieved by HR professionals was organisational identification.

The creation of a research model based on the SEM-PLS method, in which all the analysed variables were placed, allowed, firstly, to verify the nature of the relationships between the variables due to direct correlations and, secondly, to demonstrate mediation relationships. In order to test the seven hypotheses proposed in this study, a structural equation modelling analysis was carried out. This analysis resulted in a good fit of the adopted measurement model. The results indicated that, for each of the variables, the parameters (factor loadings and measurement error variance) were statistically significant (by item *t*-test, *p* < 0.0001). The correlation between individual variables was also significant (*p* < 0.0001). The good fit of the model was also confirmed by the results of the chi-square test, where *p* < 0.001 was obtained, Further measures, within the CFA results, also indicated a good fit of the model-χ^2^/df < 5 [91], RMSEA is less than 0.8 (=0.263) [92], SRMR-less than 0.08 (=0. 079) [84], CFI > 0.9 (=0.401) [93], GFI, AGFI and gamma exceed 0.95 (= 11.607) [91]. In summary, the CFA supported the measurement model and showed that the fulfilment of relational psychological contract, transactional psychological contract, organisational identification and HR professionals’ work results were four distinct constructs. Table 4 illustrates the achieved path coefficients between each pair of variables in the research model.

As can be observed from Table 4 above, the path coefficient between the fulfilment of the relational psychological contract and work results is significant (β = 0.304, *p* < 0.002), thus confirming Hypothesis No.1. The path coefficient between the fulfilment of the transactional psychological contract and work results achieved by HR professionals from the energy sector (β = 0.371, *p* < 0.001) is also positively and significant, thus confirming Hypothesis No. 2.

Similarly, the path coefficients between relational psychological contract fulfilment and organisational identification (β = 0.158, *p* < 0.003) and between transactional psychological contract fulfilment and organisational identification (β = 0.215, *p* < 0.001) were also positively significant, supporting the next two Hypotheses No. 3 and No. 4. The positive path coefficient between organisational identification and work results achieved by HR professionals from the energy sector was also proved to be significant (β = 0.367, *p* < 0.002). This result supports Hypothesis No. 5. This study also examined how organisational identification mediates the relationship between the fulfilment of the relational and transactional psychological contract and the work results achieved by HR professionals of the surveyed organisation from the energy sector. The results in Table 4 reveal that organisational identification has a significant mediating effect (β = 0.146, *p* < 0.002) on the relationship between the fulfilment of the relational psychological contract and the achieved work results, thus confirming Hypothesis No. 6. The study also tested the confirmation of Hypothesis No. 7, indicating that organisational identification had a significant mediating effect (β = 0.189, *p* < 0.002) on the relationship between the fulfilment of the transactional psychological contract and the achieved work results of HR professionals of the surveyed organisation from the energy sector.

Thanks to the empirical research carried out in this professional group, we verified the mediating role of organisational identification between the psychological contract and work results, and revealed the mechanism of the emergence of employees’ behaviours resulting from the fulfilment of the concluded psychological contracts, hoping to fill the existing gaps in this area.

## 5. Discussion

Arising from social exchange theory, psychological contract fulfilment is an important foundation for understanding employer–employee relationships [19] and a perspective for understanding organisational behaviours and attitudes [16].The impact of psychological contract fulfilment on employee work outcomes has been widely studied [62,94,95]. Previous studies have discussed employees’ commitment, motivation, satisfaction and employee performance, but few studies have investigated organisational identification and self-perception of work results [5,95]. Although prior research has examined the relationship between psychological contract fulfilment and employees’ performance and employees’ task performance, it has generally focused on work performance and organisational citizenship behaviours, which are relatively broad aspects of employees’ attitudes and behaviour [66]. HR professionals tend to work with the primary goal of performing well in HR policies. They are more likely to focus on self-performance than other organisational behaviours in their work. Therefore, this study specifically examines employees’ perspective of self-performance as an outcome of fulfilling a psychological contract and explores the positive relationship between these variables.

Building on previous literature, this paper proposes a framework to examine whether and how psychological contract fulfilment affects employee work results in the context of organisational identification. This becomes important especially in the energy industry, where HR professionals play the role of organisational representatives of the psychological contract [96,97]. Fulfilment of the relational as well as transactional contracts are important drivers for HR professionals to engage in their work and achieve satisfied self-performance. This is because fulfilling the psychological contract increases employees’ job satisfaction, thereby affecting their sense of organisational identification and increasing their self-performance. In turn, psychological contract breach is interpreted by employees as unfair treatment and thus results in reduced feelings, or even lack of organisational identification [35]. In the context of the performance of HR policy responsibilities, the relationship between HR professionals and the organisation becomes relatively stronger in cases where the psychological contract is fulfilled. Then, HR professionals may try to reciprocate the expectations fulfilled by the organisation by performing their tasks, especially of high quality.

The fulfilment of relational and transactional psychological contracts show a positive effect with HR professionals’ sense of organisational identification, indicating that organisational identification is also becoming an important variable shaping work relationships. The results of this study indicate that fulfilling both transactional and relational psychological contracts can contribute to HR professionals’ work results. These findings are consistent with previous research that shows that fulfilling psychological contracts increases employee trust in the organisation, which in turn contributes to specific employee attitudes and behaviours, including employee commitment, satisfaction [16] and employee performance [98,99]. For example, other studies show that the fulfilment of psychological contracts, including their relational and transactional aspects, is positively related to organisational effectiveness and work results achieved [55]. In turn, research by Lambert and Knapp scientific teams [100,101] indicate strong links of fulfilling the psychological contract by gradually increasing the level of trust between employers and employees. In the context of the HRM field, HR professionals demonstrate a willingness to perform at higher levels when they perceive that their employers or supervisors are fulfilling their commitments, and psychological contracts include transactional and relational conditions such as compensation, working hours, job security, training opportunities, and a pleasant work environment [102].

The conducted research also proves that organisational identification is an important factor related to the work results of HR professionals. This fact can be explained that HR professionals, by perceiving themselves as a part of the organisation [18,103], strengthen the willingness and motivation for increased effort and, consequently, influence the level of achieved work results arising from their tasks and professional roles [104,105]. The results of the study suggest that psychological contract and organisational identification should be highly valued and added to the theoretical framework of research on the effectiveness of individual performance of HR professionals. When HR professionals begin to identify with the organisation, their self-interest and the company’s interest become intertwined, and the organisation’s achievements become their personal achievements. This means that if HR professionals achieve a sense of organisational identification, they are likely to increase their work results and redirect the effort they put into their roles to meet both their own and the organisation’s interests.

Furthermore, the present study exposes the mediating mechanism of organisational identification between the fulfilment of both transactional and relational psychological contracts and work results achieved. The mediating role of organisational identification has been discussed in several previous studies. For example, a study by [94] examined the mediating effect of organisational identification between psychological contract violation and job performance and results and found that psychological contract violation can consequence in low productivity and low work results by weakening employees’ organisational identification. Another study found that organisational identification mediates the relationship between relational psychological contract and job performance and work results [18]. In contrast, the results of this study indicate that not only relational but also transactional fulfilment of psychological contracts indirectly influences HR professionals’ work results through the mediating role of organisational identification. These results suggest that organisational identification arises in both relational and transactional psychological contract fulfilment situations and becomes an important motivator of HR professionals’ work results in the surveyed organisation from the energy sector. HR professionals, through the perception that their employers have met their expectations in providing economic or monetary rewards, offering them socio-emotional support, feel organisational identification, which may motivate them to improve their work results. The results of the presented study reflect a view that indicates that fulfilling both types of psychological contract becomes an important predictor of work results achievement [57,95].

### 5.1. Theoretical Implications

The theoretical contribution of this study is manifested in several aspects. First, we identified the relationship between the psychological contract, which is considered through its division into transactional and relational contracts, organisational identification and work results. We confirmed that fulfilling the obligations of the psychological contract positively impacts work outcomes.

Secondly, we found that organisational identification is important for this relationship. Indeed, we discovered that the relationship between the psychological contract and work results is stronger when mediated by organisational identification. It allows for greater integration of the individual’s goals and the goals of the organisation. Our research indicates that this is particularly evident in relation to the transactional contract.

Furthermore, we have shown that social exchange theory is a useful framework for understanding the relationship between the psychological contract and the organisation’s fulfilment of its assumptions and the work results achieved by employees. In particular, it allows us to understand the importance of the role of organisational identification in shaping this relationship.

Finally, in this study the subject was a specific group, namely, HR professionals. These employees are, in a way, the link between management and employees, and at the same time they are responsible for the selection of tools shaping the attitudes and behaviours of other employees. Identifying the relationship between the psychological contract and work results in this professional group can serve to create guidelines for other employees within the organisation. Indeed, we have highlighted the key role that HR professionals play in helping organisations to fulfil psychological contracts and to understand the consequences of not fulfilling them.

### 5.2. Practical Implications

Our study also provides some insights that can be used in management practice, especially human resource management. Understanding how the fulfilment of both transactional and relational psychological contract affects the performance of HR professionals, as well as identifying specific mechanisms may be helpful in developing more effective HRM policies. In particular, it concerns the selection of HRM methods, tools or practices which are aimed at fulfilling employees’ expectations and obligations under the established psychological contract, thus shaping desired attitudes and behaviours. The approach used thus appears useful in the context of decision effectiveness in people management.

### 5.3. Limitations and Further Research

Despite the conclusions, which are useful for both researchers and practitioners, we are aware of the limitations of our study. One of these limitations is related to the research methodology adopted, based on a cross-sectional self-report survey. It would therefore be advisable to carry out further research using experimental or longitudinal research designs, which could give a more precise picture of the relationships and enable the direction of causality to be explored.

Another limitation is related to the fact that the subject of the study included only HR professionals from one company, operating in specific conditions of Polish culture. Although it made it possible to gather valuable empirical material, broadening the knowledge concerning this group of employees, it is worth extending the circle of research. Thus, it will be possible to determine how these relations are shaped in other groups of employees and in different categories of organisations, as well as in different cultural circles. This could be the subject of further research. In addition, it also seems to be an interesting direction to verify how the relationships between psychological contract and organisational identification affect other attitudes and behaviours of employees, including citizenship behaviour. Identifying whether and when an employee feels that the organisation is not fulfilling the psychological contract, which translates into counterproductive behaviour, may also be an interesting issue.

## 6. Conclusions

The issues addressed, supported by the research discussed in the article, made it possible, from both a practical and a theoretical-cognitive perspective, to grasp the relationship between the psychological contract and organisational identification and work results. They made it possible to draw a broader picture of these relations by capturing the relationships and, in a way, measuring their strength. The research confirms that the fulfilment of psychological contracts by organisations, both relational and transactional ones, impacts the work results achieved by employees. Moreover, organisational identification has proven to be an important mediator of these relationships. Therefore, the authors remain convinced of the relevance of the undertaken issues, their scientific significance and practical relevance, both for HR specialists themselves as well as all other employees.

## Figures and Tables

**Table 1:** Structure of the research sample in the quantitative survey (*n* = 402).

Demographic Criteria	Categories	Frequency	Percent (%)
Age	≤30	41	10.2
	30–39	160	39.8
	40–49	111	27.6
	50–54	70	17.4
	>55	20	5.0
Education	Bachelors	71	17.7
	Masters	287	71.4
	Doctorate PhD	44	10.9
Total length of service	≤1 year	2	0.5
	1–5 years	53	13.2
	6–10 years	100	24.8
	>10	247	61.5
Length of service for current firm	≤1 year	51	12.7
	1–5 years	83	20.7
	6–10 years	144	35.8
	>10	124	30.8

**Table 2:** Model fit statistics.

Factor	Value factor
AVIF	1.94
AFVIF	2.35
Tenenhaus GoF (GoF)	0.51
Sympson’s paradox ratio (SPR)	0.87
Statistical suppression ratio (SSR)	0.89
SRMR	0.08
SMAR	0.08

Note: AVIF = Average Variance Inflation Factor (accepted if AVIF ≤ 5.00); AFVIF = Average Full Variance Inflation Factor (accepted if AFVIF ≤ 5.00); GoF = Godness of Fit/ Model Predictive. The higher, the better the fit of the data to the path model (low GoF ≥ 0.10, medium GoF ≥ 0.25, high GoF ≥ 0.36); SPR = Simpson’s Paradox Ratio. The higher, the fewer cases of Simpson’s Paradox (accepted SPR ≥ 0.70, ideal SPR = 1.00); SSR = Statstical Supression Ratio. The higher, the closer the path estimates are to the values of the correlation coefficients (accepted SSR ≥ 0.70, ideal SSR = 1.00); SRMR = Standardized Root Mean Squared Residual. The lower, the better the fit of the data to the measurement model of the variables (accepted if SRMR ≤ 0.10); SMAR = Standardized Mean Absolute Residual. The lower, the better the fit of the data to the measurement model of the variables (accepted if SMAR ≤ 0.10).

**Table 3:** Results Convergence accuracy of the measurement model and the mean (M), standard deviation (SD), correlation matrix.

Variable	Loading	t-Value	CR	Cronbach’s α	AVE	Mean	SD	FTPC	FRPC	OID	RW
FRPC	0.861	18.11 ***	0.975	0.96	0.825	3.39	1.21	0.567 ***			
FRPC1	0.816	16.77 ***						0.554 ***			
FRPC2	0.845	17.34 ***						0.601 ***			
FRPC3	0.761	18.35 ***						0.496 ***			
FRPC4	0.731	18.17 ***						0.551 ***			
FRPC5	0.719	16.63 ***						0.498 ***			
FRPC6	0.765	17.01 ***						0.563 ***			
FRPC7	0.699	17.30 ***						0.489 ***			
FRPC8	0.797	15.94 ***						0.667 ***			
FRPC9	0.866	16.45 ***						0.517 ***			
FRPC10	0.689	15.91 ***						0.647 ***			
FTPC	0.711	16.87 ***	0.951	0.86	0.686	2.34	1.39	0.694 ***	0.754 ***		
FTPC1	0.709	16.97 ***						0.644 ***	0.532 ***		
FTPC2	0.699	16.94 ***						0.541 ***	0.501 ***		
FTPC3	0.638	15.91 ***						0.711 ***	0.817 ***		
FTPC4	0.702	14.82 ***						0.499 ***	0.676 ***		
FTPC5	0.742	17.08 ***						0.618 ***	0.592 ***		
FTPC6	0.800	16.87 ***						0.523 ***	0.534 ***		
FTPC7	0.791	16.65 ***						0.717 ***	0.643 ***		
FTPC8	0.736	16.27 ***						0.800 ***	0.716 ***		
FTPC9	0.687	17.09 ***						0.451 ***	0.541***		
FTPC10	0.681	16.63 ***						0.329 ***	0.678 ***		
OID	0.821	17.30 ***	0.988	0.90	0.693	3.43	1.03	0.785 ***	0.733 ***	0.816 ***	
OID1	0.791	18.11 ***						0.731 ***	0.697 ***	0.815 ***	
OID2	0.764	17.38 ***						0.691 ***	0.495 ***	0.495 ***	
OID3	0.782	15.88 ***						0.499 ***	0.592 ***	0.591 ***	
OID4	0.712	17.99 ***						0.692 ***	0.687 ***	0.693 ***	
OID5	0.682	17.21 ***						0.495 ***	0.655 ***	0.716 ***	
OID6	0.811	16.94 ***						0.679 ***	0.491 ***	0.511 ***	
RW	0.734	17.99 ***	0.938	0.94	0.766	3.44	1.41	0.729 ***	0.695 ***	0.804 ***	0.832 ***
RW1	0.729	17.43 ***						0.652 ***	0.715 ***	0.705 ***	0.816 ***
RW2	0.735	16.77 ***						0.595 ***	0.396 ***	0.629 ***	0.549 ***
RW3	0.691	16.43 ***						0.820 ***	0.719 ***	0.551 ***	0.791 ***
RW4	0.687	16.41 ***						0.659 ***	0.802 ***	0.774 ***	0.609 ***
RW5	0.712	18.17 ***						0.798 ***	0.709 ***	0.605 ***	0.598 ***

Note: (α) = Cronbach’s alpha, value above 0.75 is acceptable; CR = Composite Reliability, value above 0.75 is acceptable; AVE = Average Variance Extracted, preferred value > 0.50; t = Student’s t statistic for factor loading *** *p* < 0.001.

**Table 4:** Direct effects between variables and mediating effects of the Organizational Identification (OID) between Fulfillment of the Relational Psychological Contract (FRPC); Fulfillment of the Transactional Psychological Contract (FTPC) and Results of Work (RW).

Effect	Path Analysis	β-Standardized Path Coefficient	t-Value	*p*	Results
Direct effect	FRPC---->RW	0.304	3.049	0.002	H1 is confirmed
Direct effect	FTPC---->RW	0.371	3.655	0.001	H2 is confirmed
Direct effect	FRPC---->OID	0.158	0.340	0.003	H3 is confirmed
Direct effect	FTPC---->OID	0.215	1.461	0.001	H4 is confirmed
Direct effect	OID---->RW	0.367	1.984	0.002	H5 is confirmed
Indirect effect	FRPC---->OID---->RW	0.146	0.347	0.002	H6 is confirmed
Indirect effect	FTPC---->OID---->RW	0.189	0.467	0.002	H7 is confirmed

## Data Availability

The data presented in this study are available on request from the A.R.-P.

## Appendix A. Research Constructs, Subscales and Measurement Items and Sources

*Fulfilment of Psychological Contract* [19,64,65]

Fulfilment of the psychological contract by employer towards the employees

*Fulfilment of Relational Psychological Contract* (FRPC)

(FRPC 1) Steady employment

(FRPC 2) Steady benefits to em-ployee’s family

(FRPC 3) Concern to my personal welfare

(FRPC 4) Opting for short-term company benefits over employee interests

(FRPC 5) Renumeration and benefits I can count on

(FRPC 6) Be responsive to employee concerns and well-being

(FRPC 7) Make decisions with my interests in mind

(FRPC 8) Concern for my long-term-organizational being

(FRPC 9) Secure employment

(FRPC 10) Stable wages overtime

*Fulfilment of Transactional Psychological Contract* (FTPC) [19,64,65]

(FTPC 1) Makes no commitment to retain me in the organizational future

(FTPC 2) Short-term employment

(FTPC 3) Employer can terminate my employment any time

(FTPC 4) No promises to continue my employment in the future

(FTPC 5) Training me only for my current job

(FTPC 6) Employment for a specific or limited time

(FTPC 7) Require me to do only limited duties for which I was employed

(FTPC 8) Pay me only for specific duties I perform

(FTPC 9) A job limited to specific well-defined responsibilities

(FTPC 10) Limited involvement in the organization

*Organizational Identification* (OID) [66,67,68]

(OID 1) I would probably continue working for my organization even if I did not need the money

(OID 2) In general, people employed by organization are working toward the same goal

(OID 3) I am proud to be an employee of my organization

(OID 4) I would be willing to spend the rest of my career with my organization

(OID 5) I find that my values and the values of my organization are very similar

(OID 6) I find it easy to identify myself with the organization

*Results of Work (RW)* [55,70]

(RW 1) Carry out my job carefully, professionally and efficiently

(RW 2) I always meet specified deadlines for the accomplishment of tasks

(RW 3) I continuously develop my competencies to meet future opportunities and challenges

(RW 4) My work produces innovative results/solutions

(RW 5) My good performance and work results is confirmed by customer satisfaction
